# Rhinocerebral Mucormycosis After Tooth Extraction

**DOI:** 10.1590/0037-8682-0025-2023

**Published:** 2023-07-24

**Authors:** Fatma Şimşek, Recep Yevgi, Nazım Kızıldağ

**Affiliations:** 1 Ataturk University, Faculty of Medicine, Department of Neurology, Erzurum, Turkey. Ataturk University Faculty of Medicine Department of Neurology Erzurum Turkey

**FIGURE 1:**
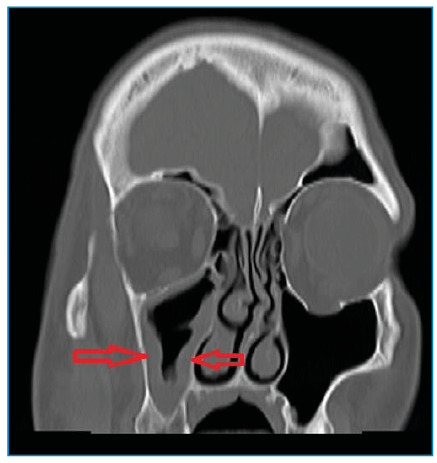
A paranasal computed tomography scan showing mucosal thickening in the right maxillary sinus.

**FIGURE 2:**
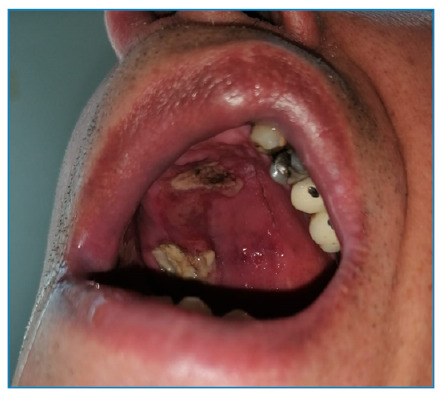
A 1.5 x 2 cm necrotic ulcerated wound observed on the palate, with the mouth shifting to the left side of the face because of right facial nerve palsy.

A 57-year-old man was presented with severe headache that had persisted for 1 week. He was diagnosed with diabetes mellitus (DM) and was followed up in the internal medicine clinic because of diabetic ketoacidosis, and he had a history of tooth extraction in the right upper jaw 10 days ago. The patient was administered with antibiotics for being diagnosed with orbital cellulitis. A paranasal computed tomography (CT) scan performed before treatment revealed mucosal thickening in the maxillary sinus (Figure 1). Neurological examination revealed ptosis, total ophthalmoplegia, the absence of light reflex in the right eye, facial paralysis, and a faint right nasolabial groove. Cranial nerve examination revealed a 1.5 x 2 cm necrotic ulcerated wound on the palate (Figure 2). We considered rhinocerebral mucormycosis as the patient had diabetic ketoacidosis, multiple cranial neuropathies, sinus involvement on radiological imaging, and necrotic intraoral wounds. He was administered with amphotericin B, and the infection site was debrided. Histopathological examination confirmed the diagnosis. However, the patient passed away after 2 months of follow-up in the intensive care unit. The symptom triad for mucormycosis comprises uncontrolled DM, periorbital infection, and meningoencephalitis^1^. Rare cases of polymicrobial rhinocerebral infection leading to mucormycosis after tooth extraction have been reported in literature^2^. Therefore, it is crucial to rule out mucormycosis in patients with uncontrolled DM and mild inflammatory findings on CT scans, who present with severe headaches after tooth extraction.
